# Measurements of radiocesium in animals, plants and fungi in Svalbard after the Fukushima Daiichi nuclear power plant disaster

**DOI:** 10.1016/j.heliyon.2019.e03051

**Published:** 2019-12-24

**Authors:** Yoshihiro Mezaki, Shigeaki Kato, Osamu Nishikawa, Isao Takashima, Masaharu Tsubokura, Haruka Minowa, Tadashi Asakura, Tomokazu Matsuura, Haruki Senoo

**Affiliations:** a Department of Laboratory Medicine, The Jikei University School of Medicine, Tokyo, Japan; b Center for Regional Cooperation, Iwaki Meisei University, Fukushima, Japan; c Department of Earth Resource Science, Akita University Graduate School of International Resource Sciences, Akita, Japan; d Akita University, Akita, Japan; e Department of Radiation Protection, Soma Central Hospital, Fukushima, Japan; f Radioisotope Research Facilities, The Jikei University School of Medicine, Tokyo, Japan; g Department of Cell Biology and Morphology, Akita University Graduate School of Medicine, Akita, Japan; h Health Center for the Elderly Kurakake-no-sato, Social Welfare Corporation Keijinkai, Akita, Japan

**Keywords:** Environmental science, Environmental radioactivity, Water pollution, Bioaccumulation, Aquatic biology, Quality of life, Svalbard, Arctic, Cesium, Radioactivity, Fukushima Daiichi nuclear power plant

## Abstract

An earthquake struck the eastern part of Japan on March 11, 2011. The Fukushima Daiichi nuclear power plant was severely damaged by the earthquake and subsequent tsunami, leading to the emission of large amounts of radioactive pollutants, including ^134^Cs and ^137^Cs, into the environment. From August 23 to September 1 in 2011, and from August 27 to September 4 in 2013, we collected samples of animals, plants, fungi and lichens from Svalbard, Norway and measured the radioactivity of ^134^Cs and ^137^Cs contained in the samples. Though no radioactivity of ^134^Cs, which has a half-life of approximately 2 years, was observed, radioactivity of ^137^Cs, which has a half-life of approximately 30 years, was observed in some samples of lichens and fungi. We failed to detect the radioactivity of ^134^Cs in any of the samples we collected, therefore, it was impossible to say clearly that the radioactivity is derived from Fukushima or not. Nevertheless, the radioactivity data documented in this report are a useful reference for the future surveys of radioactivity within the Arctic.

## 1 Introduction

Environmental pollutants are produced mainly by human activities and therefore, the primary sources of environmental pollutants are highly urbanized areas. However, environmental pollutants do not remain at the production site, but rather spread worldwide and affect the environment globally. Because pollutants frequently collect in the polar regions, these areas provide an accurate representation of the global impact of environmental pollutants [1, 2]. The polar regions have been proven as ‘sinks’ of environmental pollutants. Environmental pollutants can spread via many routes, including atmospheric distribution, ocean currents, and terrestrial waterways [3]. Pollutants also accumulate in organisms and spread through the food chain [4]. Environmental pollutants include herbicides, antiseptics, plastics, detergents, chlorofluorocarbons, dioxins, medical disposals, radioactive waste and others. In the case of radiocesium, no clear accumulation of this radionuclide has been reported. Only lichen could accumulate radiocesium and because reindeer eats it thus reindeer meat concentration is high. Among the environmental pollutants, radioactive waste is of particular concern, as it persists for many years, and is a health threat for organisms [5].

Depending upon the cause of emission, different types of radioactive pollutants are emitted into the environment. Atomic bombs on nuclear weapon testings release predominately ^235^U, ^239^Pu and ^240^Pu. Furthermore, the nuclear fission of the emitted ^235^U generates small amounts of ^137^Cs, ^90^Sr, ^133^Xe and ^131^I. On the other hand, nuclear power plant accidents mainly lead to emission of ^137^Cs, ^90^Sr, ^133^Xe and ^131^I. Additionally, stable isotope ^133^Cs catches a neutron to become radioactive ^134^Cs, a characteristic isotope for nuclear power plant accidents.

Among these radioactive pollutants, ^134^Cs and ^137^Cs are primarily monitored after nuclear power plant accidents, as cesium is easily incorporated into organisms in place of potassium [6]. The half-life of ^134^Cs is about 2 years, while ^137^Cs has a half-life of about 30 years, and thus has long-term effects on humans and the environment. It is also very useful to assess the ratio of ^137^Cs–^134^Cs, as the ratios are known to be highly correlated to the duration of operation of each nuclear power plant [7]. Therefore, this ratio can be used to predict the time of radioactive emission if the source of radioactivity is evident, or conversely to predict the source of radioactivity if the time of emission is evident.

Because cesium behaves similarly to potassium within organisms [8], enrichment of radiocesium via the food chain should be considered, especially in Arctic regions, which have a limited number of species and simple food web structure, leading to strong enrichment of radiocesium in animals at the top of the food chain. For example, polar bears, one of the top predators in Arctic regions, accumulate radiocesium as they are at the top of the food chain. Conversely, reindeers solely eat lichens, which are known to take up large amounts of radiocesium. Therefore, reindeers that eat solely lichens in the Arctic also take up large amounts of radiocesium through the lichens. Ultimately, the Sami people, who live in the Arctic and eat reindeers, take-up considerable amounts of radiocesium, which could present a significant health threat [9, 10].

An earthquake of magnitude 9.0 struck the east coast of Japan on March 11, 2011. The Fukushima Daiichi nuclear power plant was severely damaged by the earthquake and subsequent tsunami, leading to the emission of large amounts of radioactive pollutants, including ^134^Cs and ^137^Cs, into the environment [11]. The details of the accident are precisely documented in the report made by United Nations Scientific Committee on the Effects of Atomic Radiation (UNSCEAR) [12]. This degree of radioactive pollution has not occurred since the Chernobyl nuclear power plant accident on April 26, 1986. The amount of radiocesium discharged into the environment by the Fukushima nuclear power plant accident has been estimated to be one hundredth to one fiftieth of that emitted by nuclear weapon testing in the 1950's and 1960's [6, 13]. However, the radioactive pollutants emitted from Fukushima Daiichi nuclear power plant may expand into many regions of the earth, and this source of pollutants is of grave concern [14].

There are several potential pathways through which the radioactive pollutants from Fukushima may be distributed. One estimation reported that about 15 PBq of ^137^Cs was discharged into the air, and became fallouts which were then distributed over lands and seas [13]. The second main pathway for radioactive pollutants from Fukushima was via water used to cool down the fuel of the nuclear power plant (about 5 PBq of ^137^Cs). The cooling water used in this process entered to the sea in front of the Fukushima Daiichi nuclear power plant [13]. In addition to these two main pathways, underground water as well as rivers may also have carried small but non-negligible amount of radioactive pollutants to the sea.

Unlike the Chernobyl accident, the Fukushima Daiichi nuclear power plant accident emitted a large amount of radioactive pollutants via the ocean, so the potential spread of nuclear pollutants via ocean currents must be evaluated. Immediately after the Fukushima Daiichi nuclear power plant accident, the Helmholtz Center for Ocean Research Kiel reported a simulation for global distribution of ^137^Cs via ocean currents, predicting propagation of ^137^Cs to coastal waters of North America after about 5–6 years [15]. Actual measurements of the radioactivity of seawater collected from various areas on the earth have also been performed. On June 2013, sea water collected from the Pacific Ocean near Canada had detectable levels of radioactive pollutants which were presumed to originate in Fukushima, though in 2012 such radioactivity was not observed in that area [16]. From these reports, it appears that the radioactive pollutants reached Canada via the North Atlantic Current. However, how these radioactive pollutants spread to the other parts of the ocean and whether they ultimately reached the Arctic, which is considered a ‘sink’ for environmental pollutants, has not yet been evaluated.

We sampled the Svalbard islands of Norway for radioactive pollutants in the autumns of 2011 and 2013. The Svalbard islands are inundated by the Norwegian Current, which is a branch of the North Atlantic Current, and a potential source of many environmental pollutants. We collected several animal samples including mammals, birds, fish and several invertebrates, and widely sampled plants, fungi and lichens, and measured the radioactivity of ^134^Cs and ^137^Cs contained in those samples.

## 2 Materials and methods

### 2.1 Sample collection

We collected samples from August 23 to September 1 in 2011, and from August 27 to September 4 in 2013 in Svalbard, Norway. The protocols for animal use described in this article were approved by the Animal Research Committee of Akita University Graduate School of Medicine. All subsequent animal use adhered to the “Guidelines for Animal Experimentation” of the university. During hunting of mammals and birds, a region from pars cervicalis medullae spinalis to medulla oblongata was aimed for euthanasia of the animals. The same care was taken for euthanasia of other animals such as fish and invertebrates used in our study. Immediately after collecting animal samples, several organs were removed and cut into small pieces, including the liver, lung, heart, kidney, pancreas, thyroid gland, epididymis, digestive tract, spleen, muscle, bone, blubber and skin, when present. Samples were snap-frozen in a mixture of dry ice and ethanol (-72 °C) and preserved in dry ice in the field. When animals were small enough, they were frozen and preserved as a whole. Plant and fungus samples were collected and maintained in a mobile refrigerator at 4 °C. All samples were then transported to the University of Oslo, and subsequently transported to Akita University on dry ice. Samples were stored at -80 °C until radioactivity measurements were performed.

### 2.2 Radioactivity measurement

Sample radioactivity was measured using a coaxial germanium detector, GX1518 (relative efficiency ≥15%, Canberra Industries, Meriden, CT, USA). Samples were weighed and put into polystyrene U-8 bottles (100 mL) for the radiation measurement. We did not adopt the ashing process for getting better efficiency and decreasing the detection limit. Samples were cut and pushed against the bottom of the bottles to minimize the sample volume in the bottle. The duration of measurement for each sample was about 24 h in order to measure many samples in a short period of time. Energy and efficiency calibrations were done using activity standard gamma sources. Self-absorption correction concerning geometric setup, chemical composition and density was performed using a computer program equipped with the germanium detector. Radioactivity and detection limits were obtained using equipped software for samples collected in 2013. Because the software was not yet upgraded when the measurement of samples collected in 2011 was conducted, only the radioactivity was obtained using the equipped software, and the detection limit was calculated manually using the equation below:

$$\text{detection limit }\left[\text{counts}\right]=3\times \sqrt{\text{peak background}}$$

The consistency of the values of the detection limits obtained from 2011 and 2013 samples was confirmed by re-analyzing the 2013 data manually and comparing the calculated values with those obtained by the equipped software, resulting in almost the same values.

### 2.3 Map generation

Maps were generated using ArcGIS Explore software (Esri, Redlands, CA, USA). The GPS data recorded during sample collection were analyzed by the same software and incorporated into the map.

### 2.4 Ethical approval

All applicable international, national, and/or institutional guidelines for the care and use of animals were followed. All procedures performed in studies involving animals were in accordance with the ethical standards of the institution or practice at which the studies were conducted. The protocols for animal use described in this article were approved by Animal Research Committees of Akita University Graduate School of Medicine.

## 3 Results

### 3.1 The area and date of sample collection and radioactivity measuring

**Figure 1:**
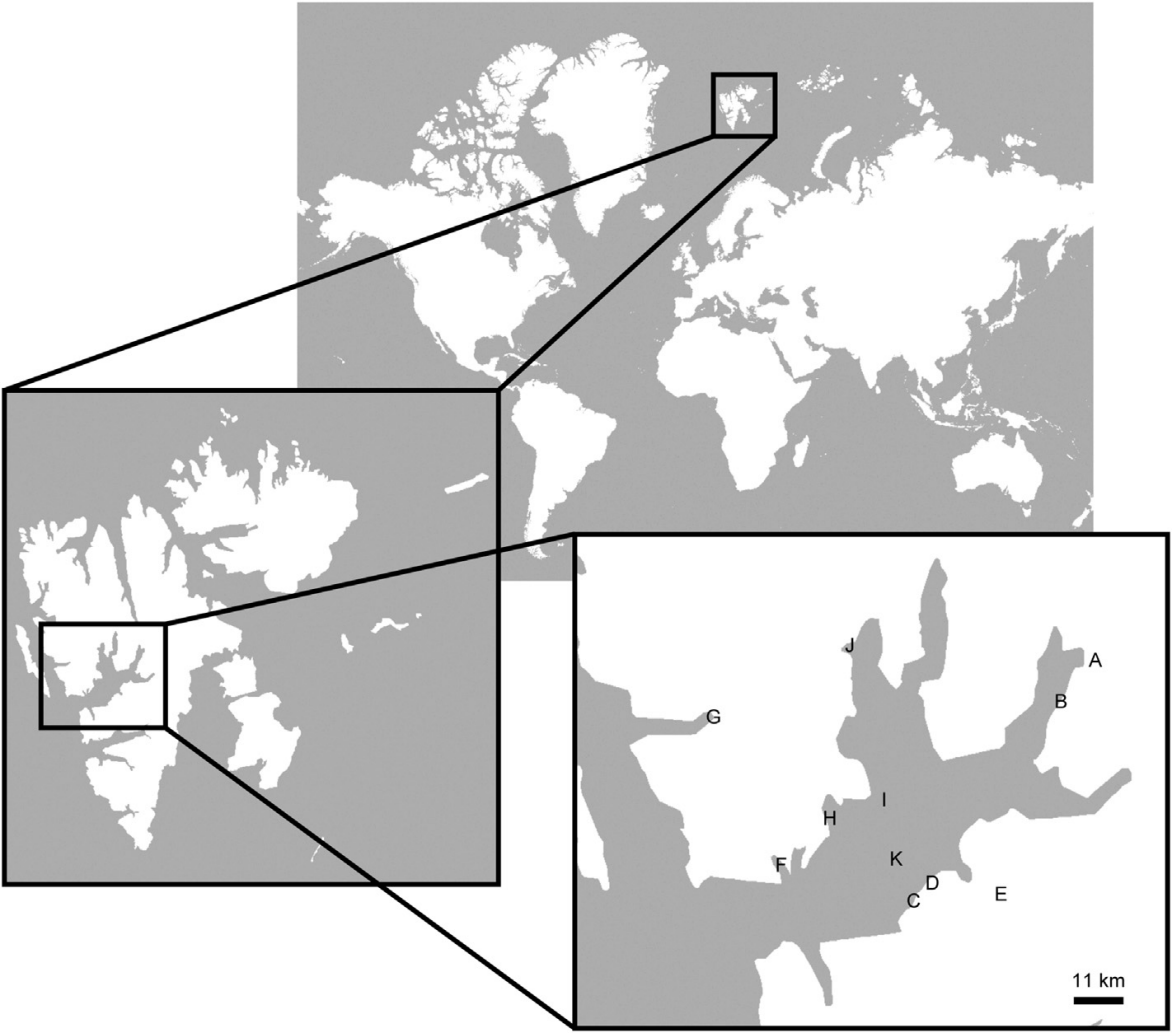
Sampling areas in Svalbard islands. The lettered points represent the sample collection sites. The maps in the figure were made with Natural Earth and are similar but not identical to the original images, and are therefore for illustrative purposes only.

The areas of sample collection in Svalbard are marked in Figure 1. Samples were collected twice during August and September in 2011, and again during August and September in 2013. Due to limited availability of logistics, all of the samples were collected at the west sides of the Svalbard, though it was estimated that a very small amount of radiocesium reached to the area [6, 17, 18]. Radioactivity of samples obtained in 2011 was measured between December 2011 and May 2012, while radioactivity of samples obtained in 2013 was measured between December 2013 and May 2014. The data is summarized in Tables 1, 2, 3, and 4. In brief, all animal samples obtained in both 2011 and 2013 had no radioactivity above detection levels of the germanium detector, GX1518 (Tables 1 and 3). Several plant samples such as grass, lichens and fungi obtained in both 2011 and 2013 had detectable ^137^Cs radioactivity (Tables 2 and 4). No ^134^Cs radioactivity was observed in the ^137^Cs-positive samples.

**Table 1:** Animal sample measurement results in 2011.

Species	Individuals	Wet weight (g)	^134^Cs (Bq/kg)	^137^Cs (Bq/kg)
Bearded seal (*Erignathus barbatus*)	#1, A	64.58	N.D. (<0.8)	N.D. (<0.7)
	#1, A	88.05	N.D. (<0.4)	N.D. (<0.4)
	#1, A	30.76	N.D. (<1.2)	N.D. (<1.2)
	#2, A	71.45	N.D. (<0.6)	N.D. (<0.6)
	#2, A	109.66	N.D. (<0.3)	N.D. (<0.3)
	#3, A	64.42	N.D. (<0.7)	N.D. (<0.6)
	#3, A	98.81	N.D. (<0.5)	N.D. (<0.4)
Ringed seal (*Phoca hispida*)	#1, A	91.89	N.D. (<0.5)	N.D. (<0.5)
	#1, A	49.50	N.D. (<0.8)	N.D. (<0.8)
	#2, B	76.41	N.D. (<0.6)	N.D. (<0.6)
	#2, B	104.03	N.D. (<0.5)	N.D. (<0.4)
	#2, B	52.31	N.D. (<0.9)	N.D. (<0.9)
Glaucous gull (*Larus hyperboreus*)	#1, C	84.68	N.D. (<0.6)	N.D. (<0.5)
	#2, C	71.73	N.D. (<0.6)	N.D. (<0.6)
	#3, C	59.43	N.D. (<0.8)	N.D. (<0.7)
	#4, C	81.77	N.D. (<0.5)	N.D. (<0.5)
	#5, C	87.37	N.D. (<0.5)	N.D. (<0.5)
	#6, C	96.25	N.D. (<0.4)	N.D. (<0.4)
	#7, C	87.18	N.D. (<0.3)	N.D. (<0.3)
	#8, C	91.52	N.D. (<0.3)	N.D. (<0.3)
	#9, C	81.31	N.D. (<0.6)	N.D. (<0.5)
	#10, C	76.27	N.D. (<0.6)	N.D. (<0.5)
Svalbard rock ptarmigan	#1, D	19.66	N.D. (<2.0)	N.D. (<1.9)
(*Lagopus muta hyperborea*)	#1, D	2.46	N.D. (<17.5)	N.D. (<15.8)
	#2, D	30.78	N.D. (<1.3)	N.D. (<1.3)
	#3, D	40.80	N.D. (<1.0)	N.D. (<1.0)
	#4, D	43.03	N.D. (<1.3)	N.D. (<1.1)
	#5, D	49.32	N.D. (<0.9)	N.D. (<0.8)
	#6, D	40.72	N.D. (<1.1)	N.D. (<1.0)
	#7, D	37.18	N.D. (<1.0)	N.D. (<0.9)
	#8, D	41.37	N.D. (<1.0)	N.D. (<1.0)
	#9, D	38.61	N.D. (<1.2)	N.D. (<1.0)
Atlantic cod (*Gadus callarias*)	#1, A	37.79	N.D. (<1.1)	N.D. (<1.0)
Pink salmon	#1, A	81.42	N.D. (<0.6)	N.D. (<0.5)
(*Oncorhynchus gorbuscha*)	#1, A	29.42	N.D. (<1.5)	N.D. (<1.3)
Swimming snail (*Limacina helicina*)	#1, A	0.73	N.D. (<42.5)	N.D. (<39.8)
Sea gooseberry (*Pleurobrachia pileus*)	#1, A	17.61	N.D. (<2.3)	N.D. (<2.0)

Letters after individual numbers represent the sample collection site (see Figure 1).

N.D. represents “not detected.”

The detection limit is presented in brackets.

**Table 2:** Plant and fungus sample measurement results in 2011.

Species	Individuals	Wet weight (g)	^134^Cs (Bq/kg)	^137^Cs (Bq/kg)
Lichen (*Flavocetraria cucullata*)	#1, B	14.86	N.D. (<2.5)	53.2 ± 5.5 (<2.4)
Lichen (*Lobaria linita*)	#1, E	2.96	N.D. (<12.3)	N.D. (<11.9)
Lichen (*Ochrolechia frigida*)	#1, E	3.30	N.D. (<11.7)	N.D. (<9.9)
Lichen (*Usnea sphacelata*)	#1, E	2.44	N.D. (<16.2)	46.4 ± 13.4 (<14.0)
Lichen (*Cladonia rangiferina*)	#1, B	20.44	N.D. (<2.0)	4.6 ± 1.6 (<1.8)
Lichen (*Cladonia arbuscula*)	#1, B	9.60	N.D. (<4.1)	5.4 ± 2.2 (<3.7)
Mushroom	#1, C	28.57	N.D. (<1.5)	N.D. (<1.3)

Letters after individual numbers represent the sample collection site (see Figure 1).

N.D. represents “not detected.”

The detection limit is presented in brackets.

The error represents 3-sigma of counting statistics.

**Table 3:** Animal sample measurement results in 2013.

Species	Individuals	Wet weight (g)	^134^Cs (Bq/kg)	^137^Cs (Bq/kg)
Bearded seal (*Erignathus barbatus*)	#1, G	465.0	N.D. (<0.2)	N.D. (<0.3)
	#2, G	311.0	N.D. (<0.3)	N.D. (<0.3)
	#3, G	182.1	N.D. (<0.6)	N.D. (<0.8)
Ringed seal (*Phoca hispida*)	#1, F	197.5	N.D. (<0.7)	N.D. (<0.8)
	#2, F	165.0	N.D. (<0.8)	N.D. (<1.0)
	#3, J	131.7	N.D. (<1.1)	N.D. (<1.2)
Glaucous gull (*Larus hyperboreus*)	#1, F	36.2	N.D. (<2.2)	N.D. (<2.1)
	#2, F	40.9	N.D. (<0.8)	N.D. (<0.8)
	#3, F	37.5	N.D. (<0.8)	N.D. (<0.9)
	#4, F	65.5	N.D. (<0.7)	N.D. (<0.8)
	#5, F	51.8	N.D. (<1.1)	N.D. (<1.1)
	#6, H	57.5	N.D. (<0.8)	N.D. (<1.0)
	#7, J	39.9	N.D. (<0.6)	N.D. (<0.7)
	#8, J	56.0	N.D. (<0.9)	N.D. (<0.9)
	#9, C	51.1	N.D. (<2.0)	N.D. (<2.0)
	#10, C	53.7	N.D. (<1.1)	N.D. (<1.1)
	#11, C	51.5	N.D. (<0.9)	N.D. (<0.9)
	#12, C	52.1	N.D. (<0.6)	N.D. (<0.6)
	#13, C	48.6	N.D. (<1.1)	N.D. (<1.1)
	#14, C	39.6	N.D. (<0.9)	N.D. (<0.9)
Svalbard rock ptarmigan	#1, D	19.1	N.D. (<2.4)	N.D. (<2.4)
(*Lagopus muta hyperborea*)	#2, D	13.8	N.D. (<1.9)	N.D. (<2.1)
	#3, D	16.4	N.D. (<1.5)	N.D. (<1.4)
	#4, D	19.4	N.D. (<3.3)	N.D. (<3.1)
	#5, D	18.6	N.D. (<1.4)	N.D. (<1.3)
	#6, D	16.0	N.D. (<2.0)	N.D. (<2.1)
	#7, D	17.9	N.D. (<1.3)	N.D. (<1.2)
	#8, D	23.6	N.D. (<1.4)	N.D. (<1.3)
	#9, D	18.6	N.D. (<2.7)	N.D. (<2.7)
	#10, D	21.8	N.D. (<3.1)	N.D. (<2.8)
Atlantic puffin (*Fratercula arctica*)	#1, F	30.6	N.D. (<1.2)	N.D. (<1.3)
	#2, F	24.1	N.D. (<2.5)	N.D. (<2.6)
	#3, F	17.6	N.D. (<1.1)	N.D. (<1.0)
	#4, F	15.1	N.D. (<2.1)	N.D. (<1.9)
	#5, F	15.4	N.D. (<2.3)	N.D. (<2.3)
	#6, F	13.9	N.D. (<2.3)	N.D. (<2.2)
	#7, K	16.9	N.D. (<2.0)	N.D. (<1.9)
	#8, K	19.0	N.D. (<1.8)	N.D. (<2.0)
	#9, K	20.1	N.D. (<1.9)	N.D. (<1.7)
	#10, K	22.6	N.D. (<1.6)	N.D. (<1.8)
Northern fulmar (*Fulmarus glacialis*)	#1, F	22.7	N.D. (<1.5)	N.D. (<1.6)
	#2, F	25.3	N.D. (<2.0)	N.D. (<1.8)
	#3, F	24.2	N.D. (<1.0)	N.D. (<1.4)
	#4, F	21.8	N.D. (<1.2)	N.D. (<1.2)
	#5, F	23.3	N.D. (<1.7)	N.D. (<1.6)
	#6, F	20.5	N.D. (<1.6)	N.D. (<1.6)
	#7, F	22.4	N.D. (<1.6)	N.D. (<1.5)
	#8, F	20.7	N.D. (<1.3)	N.D. (<1.2)
	#9, F	26.4	N.D. (<1.4)	N.D. (<1.4)
	#10, F	24.8	N.D. (<0.9)	N.D. (<1.0)
Atlantic cod (*Gadus callarias*)	#1, I	33.0	N.D. (<1.2)	N.D. (<1.4)
	#2, I	13.8	N.D. (<2.5)	N.D. (<2.2)
	#3, I	18.7	N.D. (<1.8)	N.D. (<1.9)
	#4, I	12.5	N.D. (<2.6)	N.D. (<2.6)
	#5, I	25.7	N.D. (<1.1)	N.D. (<1.2)
	#6, I	32.4	N.D. (<1.1)	N.D. (<1.3)
	#7, I	20.5	N.D. (<1.2)	N.D. (<1.2)
Arctic char (*Salvelinus alpinus*)	#1, H	26.1	N.D. (<1.4)	N.D. (<1.4)
	#2, H	15.9	N.D. (<1.8)	N.D. (<1.9)
	#3, H	19.4	N.D. (<1.5)	N.D. (<1.7)
Shorthorn sculpin	#1, F	5.9	N.D. (<5.3)	N.D. (<5.2)
(*Myoxocephalus scorpius*)	#2, F	10.2	N.D. (<1.6)	N.D. (<1.6)
	#3, F	8.0	N.D. (<3.7)	N.D. (<3.8)
	#4, F	7.2	N.D. (<4.1)	N.D. (<4.1)
	#5, H	14.7	N.D. (<2.1)	N.D. (<2.0)
Jellyfish	#1, F	22.2	N.D. (<1.1)	N.D. (<1.2)
	#2, H	75.2	N.D. (<0.7)	N.D. (<0.9)

Letters after individual numbers represent the sample collection site (see Figure 1).

N.D. represents “not detected.”

The detection limit is presented in brackets.

**Table 4:** Plant and fungus sample measurement results in 2013.

Species	Individuals	Wet weight (g)	^134^Cs (Bq/kg)	^137^Cs (Bq/kg)
Grass (*Saxifraga* sp. A)	#1, H	5.8	N.D. (<3.8)	N.D. (<3.7)
	#2, H	2.1	N.D. (<13.7)	N.D. (<12.5)
Grass (*Saxifraga* sp. B)	#1, H	34.1	N.D. (<1.8)	13.3 ± 2.1 (<1.9)
Grass (*Saxifraga* sp. C)	#1, H	4.6	N.D. (<4.9)	18.3 ± 3.6 (<4.9)
	#2, H	9.0	N.D. (<2.8)	17.0 ± 2.6 (<2.9)
Grass (*Saxifraga* sp. D)	#1, H	37.4	N.D. (<1.4)	4.9 ± 0.8 (<1.3)
Grass (*Carex* sp.)	#1, H	22.2	N.D. (<1.3)	7.5 ± 1.1 (<1.3)
Grass (*Cerastium arcticum*)	#1, H	7.9	N.D. (<4.6)	23.7 ± 4.3 (<5.1)
Grass (*Luzula confusa*)	#1, H	2.3	N.D. (<12.4)	N.D. (<12.0)
Seaweed (*Laminariaceae* spp.)	#1, F	70.3	N.D. (<1.1)	N.D. (<1.2)
	#2, F	81.8	N.D. (<1.0)	N.D. (<1.1)
	#3, F	4.1	N.D. (<6.7)	N.D. (<6.2)
	#4, F	109.7	N.D. (<0.8)	N.D. (<0.9)
	#5, H	72.7	N.D. (<0.8)	N.D. (<0.9)
Seaweed (*Undaria* spp.)	#1, F	25.5	N.D. (<1.6)	N.D. (<1.8)
	#2, F	25.0	N.D. (<1.4)	N.D. (<1.4)
	#3, F	102.6	N.D. (<0.6)	N.D. (<0.7)
	#4, G	13.2	N.D. (<2.6)	N.D. (<2.4)
	#5, G	3.6	N.D. (<7.3)	N.D. (<7.5)
	#6, H	51.3	N.D. (<1.2)	N.D. (<1.5)
Lichen (*Flavocetraria cucullata*)	#1, H	40.3	N.D. (<1.1)	20.2 ± 1.7 (<1.2)
	#2, H	9.2	N.D. (<3.3)	31.6 ± 3.8 (<3.1)
	#3, H	38.7	N.D. (<1.5)	N.D. (<1.7)
	#4, H	6.7	N.D. (<5.4)	36.1 ± 5.7 (<5.5)
Lichen (*Neuropogon shaceratus*)	#1, H	38.3	N.D. (<1.8)	11.9 ± 1.7 (<1.7)
	#2, H	1.3	N.D. (<23.8)	N.D. (<21.8)
Mushroom (*Arrhenia lobata*)	#1, H	1.5	N.D. (<15.4)	N.D. (<14.8)
Mushroom (*Hebeloma polare*)	#1, H	2.0	N.D. (<14.6)	N.D. (<13.5)
Mushroom (*Lactarius lanceolatus*)	#1, H	6.0	N.D. (<4.6)	N.D. (<4.6)
Mushroom (*Russula nana*)	#1, H	4.2	N.D. (<5.5)	N.D. (<5.7)
Mushroom (*Russula* spp.)	#1, H	9.9	N.D. (<3.3)	N.D. (<3.2)
	#2, H	2.3	N.D. (<11.7)	36.2 ± 7.9 (<11.0)
	#3, H	1.0	N.D. (<21.6)	N.D. (<22.5)
	#4, H	5.5	N.D. (<5.8)	10.1 ± 3.1 (<5.5)
	#5, H	4.0	N.D. (<7.6)	N.D. (<7.6)
	#6, H	2.5	N.D. (<11.5)	N.D. (<10.4)

Letters after individual numbers represent the sample collection site (see Figure 1).

N.D. represents “not detected.”

The detection limit is presented in brackets.

The error represents 3-sigma of counting statistics.

### 3.2 Estimated origin of radioactivity observed in some samples in 2011 and 2013

We next sought to determine if the radioactivity observed in lichen, plant and fungus samples was likely to be derived from the Fukushima Daiichi nuclear power plant. Unfortunately, we failed to detect the radioactivity of ^134^Cs in any of the samples we collected, which is necessary to determine whether the radioactivity is derived from Fukushima or not.

## 4 Discussion

After the Fukushima Daiichi nuclear power plant accident on March 11, 2011, a large amount of radioactive pollutants was emitted into the environment. Radioactive pollutants that were emitted directly to the ocean, drained into the ocean via rivers or underground waterways, are all predicted to spread globally via ocean currents. The radioactive pollutants thus ultimately reach the Arctic. We have collected samples of animals, plants, fungi and lichens from Svalbard, Norway in the Arctic. The samples were collected twice, during the autumns of 2011 and 2013. Radioactivity of ^134^Cs and ^137^Cs was measured using a germanium semiconductor detector. No radioactivity of ^134^Cs, which has a half-life of approximately 2 years, was observed. However, radioactivity of ^137^Cs, which has a half-life of approximately 30 years, was observed in some samples of plants, lichens and fungi. Our data, especially the radioactivity of ^137^Cs for fish and seaweed, are in good agreement with previous measurements conducted by Norwegian National Monitoring Programme (NRPA) [19], presumably due to relatively uniform distribution of ^137^Cs radioactivity in seawater.

Both ^134^Cs and ^137^Cs are emitted from nuclear power plant accidents, but the composition of these two radioactive materials is known to be diverse. The amount of ^134^Cs emitted from a nuclear power plant changes depending on the duration of the operation time of the nuclear power plant. It is known that the relative proportion of ^134^Cs–^137^Cs discharged from the Chernobyl power plant accident was 0.55 [20,21], and that the relative proportion of ^134^Cs–^137^Cs discharged from the Fukushima Daiichi nuclear power plant was approximately 1 [22,23]. This value (the proportion of ^134^Cs over ^137^Cs) decreases gradually in a year-scale, as the half-lives of ^134^Cs and ^137^Cs are 2 and 30 years, respectively. For example, the proportion of ^134^Cs/^137^Cs emitted from Chernobyl decreases from 0.55 in 1986 to 0.000018 in 2018. Accordingly, the proportion of ^134^Cs/^137^Cs emitted from Fukushima Daiichi nuclear power plant decreases from 1 in 2011 to 0.1 in 2018. Though we have detected ^137^Cs radioactivity from some samples (Tables 2 and 4), we failed to detect the radioactivity of ^134^Cs in any of the samples we collected, which is required to calculate the proportion of ^134^Cs/^137^Cs. Therefore, it is impossible to say clearly that the radioactivity is derived from Fukushima or not.

Radioactive pollutants from atmospheric nuclear weapon testing during the 1960's and the Chernobyl nuclear power plant accident in 1986 were distributed mainly by fallouts [24]. Contrastingly, a large amount of pollutants from the Fukushima accident was distributed through ocean pollution [13], with a limited amount of distribution through fallouts [25]. It is therefore important to monitor radioactive pollutants that expand globally via ocean currents.

In addition to the distribution of radioactive pollutants via the atmosphere and ocean currents, the accumulation of radioactive pollutants via the food chain must be considered. After the Chernobyl accident, assessments of the emitted radioactive pollutants were conducted in the Northern hemisphere of the earth. Reindeer eat lichens, which take up a large amount of radiocesium, so are a likely source of radiocesium accumulation via the food chain. Accordingly, increases in the amount of radioactivity of ^134^Cs and ^137^Cs were observed in reindeer from Norway [9, 10]. Radiocesium was also detected in reindeer from northern Canada, Alaska and Greenland [26]. From the analyses of the proportion of ^134^Cs/^137^Cs in reindeer from Canada, the investigators suggested that about 20% of ^137^Cs originated from the Chernobyl accident, and that the remainder likely originated from atmospheric nuclear weapon testing. Humans such as the Sami people, who live in the Arctic area and eat reindeer, are likely to consume significant amounts of radiocesium, which is a public health risk to these populations [10]. In the present study, we collected muscles and viscera of reindeers in Svalbard and transported them to Oslo University. However, we unfortunately could not bring back these samples to Japan, as permission for the transfer of reindeer samples from Norway to Japan could not be obtained. Reindeer have a diet replete in lichens, so contamination of reindeer indicates that lichens are also contaminated by radiocesium in that region. Lichens tend to store a large amount of radiocesium as well as mushrooms, as these organisms persist for a long time in soil that is contaminated with radioactive pollutants from air and/or sea water. In our analyses, some lichens and mushrooms (fungi) had a small amount of radioactivity from ^137^Cs. Regardless of the source of this radiocesium, the radioactivity of reindeer muscle should continue to be monitored, as reindeer muscles are a human food source in the region.

After the Chernobyl accident, radioactive pollutants were mainly dispersed via the atmosphere as fallouts, while after Fukushima accidents large amount of radioactive pollutants were discharged directly into the ocean [13]. Radioactive pollutants in the ocean are slowly dispersed via ocean currents. For example, radioactive pollutants arrived on the west coast of Canada in July 2013, more than two years after the Fukushima Daiichi nuclear power plant accident [16]. This suggests that radioactive pollutants from Fukushima could ultimately reach the Arctic, but may require more dispersal time. Continued monitoring of ^134^Cs and ^137^Cs is therefore required to assess the environmental impact of the Fukushima accident. The radioactivity data documented in this report are a useful reference for the future surveys of radioactivity within the Arctic.

It is estimated that radioactivity on the west coast of North American continent reached a maximum of approximately 3–5 Bq/m^3^ from 2015-2016 [16], the estimation which was confirmed recently [27]. The estimated value is far below WHO guidelines for drinking-water quality, for which the maximum acceptable concentration of ^137^Cs is 10,000 Bq/m^3^ [28]. Therefore, if radioactive pollutants spread via ocean currents to reach the Arctic, the radioactivity would be below threatening levels for organisms. Radioactivity is naturally present on the earth and within space, and zero radiation exposure is impossible. Because excessive protection from radiation exposure presents a significant social cost [29], adequate protection from radiation exposure from the Fukushima Daiichi nuclear power plant accident is anticipated.

## Declarations

### Author contribution statement

Yoshihiro Mezaki: Performed the experiments; Analyzed and interpreted the data; Wrote the paper.

Shigeaki Kato: Analyzed and interpreted the data; Wrote the paper.

Osamu Nishikawa, Isao Takashima: Performed the experiments.

Masaharu Tsubokura, Tadashi Asakura, Tomokazu Matsuura: Analyzed and interpreted the data.

Haruka Minowa: Analyzed and interpreted the data; Contributed reagents, materials, analysis tools or data.

Haruki Senoo: Conceived and designed the experiments; Performed the experiments; Wrote the paper.

### Funding statement

This work was supported by JSPS KAKENHI (24255003 to HS, 16K00872 to YM) and the Jikei University Graduate Research Fund to TM.

### Competing interest statement

The authors declare no conflict of interest.

### Additional information

No additional information is available for this paper.
